# Methods for Spatiotemporal Analysis of Human Gait Based on Data from Depth Sensors

**DOI:** 10.3390/s23031218

**Published:** 2023-01-20

**Authors:** Jakub Wagner, Marcin Szymański, Michalina Błażkiewicz, Katarzyna Kaczmarczyk

**Affiliations:** 1 Institute of Radioelectronics and Multimedia Technology, Faculty of Electronics and Information Technology, Warsaw University of Technology, Nowowiejska 15/19, 00-665 Warsaw, Poland; 2 Chair of Physiotherapy Fundamentals, Faculty of Rehabilitation, Józef Piłsudski University of Physical Education in Warsaw, Marymoncka 34, 00-968 Warsaw, Poland

**Keywords:** gait analysis, health care, depth sensor, in-home monitoring, data processing

## Abstract

Gait analysis may serve various purposes related to health care, such as the estimation of elderly people’s risk of falling. This paper is devoted to gait analysis based on data from depth sensors which are suitable for use both at healthcare facilities and in monitoring systems dedicated to household environments. This paper is focused on the comparison of three methods for spatiotemporal gait analysis based on data from depth sensors, involving the analysis of the movement trajectories of the knees, feet, and centre of mass. The accuracy of the results obtained using those methods was assessed for different depth sensors’ viewing angles and different types of subject clothing. Data were collected using a Kinect v2 device. Five people took part in the experiments. Data from a Zebris FDM platform were used as a reference. The obtained results indicate that the viewing angle and the subject’s clothing affect the uncertainty of the estimates of spatiotemporal gait parameters, and that the method based on the trajectories of the feet yields the most information, while the method based on the trajectory of the centre of mass is the most robust.

## 1. Introduction

The typical gait requires proper functioning of the musculoskeletal and central nervous systems, which involves the ability to perform complex movement patterns and to rapidly process sensory input [1]. Thus, the analysis of human gait, aimed at detecting its abnormalities and identifying their causes, may support the development and optimisation of therapeutic procedures [2]. Useful information may be obtained by comparing a patient’s gait characteristics before and after some medical intervention [3]. Gait analysis may also be part of research activities and contribute to the design of new clinical practices [4]. Some indicators of the variability of selected spatiotemporal gait parameters are correlated with the risk of falling [5,6] and the presence and severity of Parkinson’s disease and Huntington’s disease [7].

Gait disorders typically accompany ageing-related decline in the general health status and the loss of functional mobility. The accidental falls of the elderly—of which a substantial proportion occurs during walking [8]—are one of the most common causes of bone fractures and head traumas which lead to hospitalisation [9]. The prevention of such falls—aimed at improving the quality of life of the elderly and reducing public expenditures related to health care services—has become a challenge of significant social importance because the share of the elderly in the global population is growing rapidly [10]. The results of gait analysis may serve estimation of the risk of falling [11] and may support the early diagnosis of neurodegenerative diseases [12], thus contributing to the effectiveness of fall prevention efforts.

Enabling elderly people to live independently in their households—rather than admitting them to nursing care facilities—might not only improve their quality of life but also relieve public health care institutions of some of their workloads and thus provide savings on health care expenditures [13]. Monitoring systems devoted to the household environment, capable of tracking the long-term evolution of parameters related to health status and designed to notify caretakers about the need for intervention, may enable and support elderly persons’ independent lives [14]. The development of technological solutions allowing for efficient and reliable gait analysis, applicable in monitoring systems operating in household environments, is, therefore, desirable from the social point of view [15].

**Figure 1:**
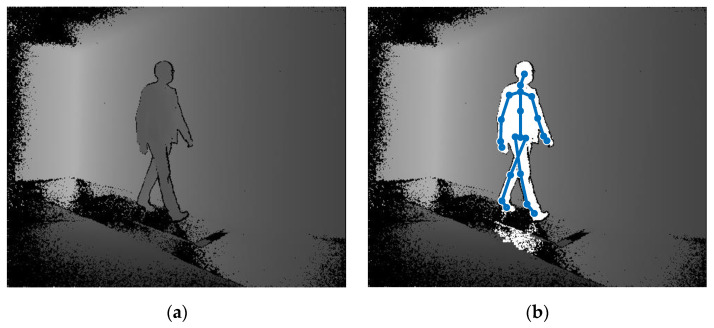
(**a**) An exemplary depth image acquired using a Kinect v2 device, in which brighter pixels indicate larger distances from the device; (**b**) The results of the detection of a human silhouette and the results of the localisation of 21 anatomical landmarks, obtained using the algorithm implemented in the Kinect v2 device.

Various techniques for clinical gait analysis have been developed, including optoelectronic motion-capture systems and platforms and treadmills equipped with force sensors [16]. Wearable sensors—such as inertial measurement units or pressure-sensing foot insoles—have also been used in commercially available gait-analysis systems and research studies [17]. Depth sensors are not yet widely used for gait analysis, but the possibility of using them for this purpose has attracted researchers’ interest for over a decade [18]. A depth sensor allows for estimating its distance from points on surfaces reflecting infrared light, thus providing data organised into so-called depth images in which each pixel represents a triplet of three-dimensional coordinates (Figure 1a). What makes such sensors particularly promising for human motion analysis is the existence of effective algorithms for detecting human silhouettes in depth images and algorithms for processing those silhouettes, to estimate the three-dimensional positions of selected anatomical landmarks, including several points along the spine, joints of the upper and lower limbs, and the centres of the head and feet (Figure 1b).

Depth sensors are much cheaper and smaller than optoelectronic motion-capture systems and tensometric platforms. They do not require any devices or markers to be worn on the body or clothes of the examined person. A functional gait analysis system may be composed of a depth sensor connected to a computer, so it can be easily installed in various spatial configurations. If the person being monitored in the home is concerned, depth sensors do not violate that person’s privacy as much as video cameras. Gait analysis systems based on depth sensors are often considered suitable for screening patients before more detailed diagnostic procedures [18]. The possibility of using depth sensors for gait analysis in monitoring systems dedicated to household environments has not yet been extensively studied, i.e., no data-processing method has been conclusively proven to yield reliable estimates of gait parameters under diverse conditions in such environments.

Despite the practical advantages of depth sensors, their application for gait analysis remains a technical challenge because of the following facts:

- estimates of the positions of anatomical landmarks obtainable using depth sensors are corrupted by non-negligible measurement uncertainty—in particular, estimates of the positions of feet [19];
- during walking, the lower limbs may occlude each other from time to time, making it difficult to accurately track the movement of both of them simultaneously;
- estimates of positions of anatomical landmarks can usually be obtained only in part of the depth sensor’s field of view, typically covering no more than 1 or 2 strides;
- the angle between the walking direction and the depth sensor’s line of sight, as well as the subject’s clothing, may affect the accuracy of localisation of anatomical landmarks.

The last point is irrelevant in the case of diagnostic procedures carried out in health care facilities where the patients can be advised to wear standard clothes and asked to walk in a specified direction. It is, nevertheless, relevant in the case of in-home monitoring systems expected to provide reliable information regardless of the monitored person’s walking direction and clothing.

Various methods for spatiotemporal gait analysis based on data from depth sensors have been proposed during the last several years. Ferraris et al. considered analysing the antero-posterior velocity of the ankles using a Kinect v2 device placed in front of the walking person [20]. Dubois et al. considered analysing the vertical oscillations of the walking person’s centre of mass using a Kinect v2 device placed at that person’s side [21] or in the corner of a room [22]. Several authors considered analysing the distance between the ankles using Kinect v2 devices placed in front of the walking person [23,24,25] or in other spatial configurations [26]. Castaño-Pino et al. considered performing the wavelet analysis of the trajectories of the ankles [27]. Albert et al. considered analysing the antero-posterior positions of the feet using Kinect v2 and Azure Kinect devices placed in front of a treadmill [28]. Vilas-Boas et al. considered analysing the distance between the ankles, the velocity of the ankles, and the shank-to-vertical angle using Kinect devices placed in front of and behind the walking person [29,30]. Atanasov and Kampel considered analysing the horizontal velocity of the walking person’s centre of mass [31]. Geerse et al. considered analysing the antero-posterior distance between the spine base and the ankles using a Kinect v2 device placed so that there was a 70° angle between its line of sight and the walking direction [32]. Auvinet et al. considered analysing the distance between the knees using a Kinect v2 device placed behind a treadmill [33]. Amini et al. considered analysing the knee joint angle and the height of the ankles using a Kinect v2 device placed in front of the walking person [34]. Xu et al. considered analysing the distance between the ankle and the hip using a Kinect v1 device placed in front of a treadmill [34,35]. Hynes et al. proposed an original algorithm for estimation of spatiotemporal gait parameters based on clustering the estimates of positions of feet [36]. Latorre et al. compared five such methods using data from a Kinect v2 device placed in front of walking persons [37]. Some authors also considered using multiple depth sensors simultaneously [38,39] or attaching depth sensors to walkers [40,41]. A systematic review of gait analysis techniques based on depth sensors can be found, e.g., in [42].

The accuracy of most of the aforementioned data-processing methods has been assessed using reference equipment such as optoelectronic motion capture systems: Vicon [20,23,28,33], Qualisys [26,29,30], or Optotrak [32,35]; tensometric platforms, i.e., GAITRite [43] or Zeno Walkway [36]; or wearable inertial measurement units [22]. However, to the best of the authors’ knowledge, only one study aimed at a systematic comparison of a subset of those methods has been published [37], and it considered only conditions in which the walking direction and the subject’s clothing can be controlled. The influence of the subject’s clothing or the angle between the depth sensor’s line of sight and the walking direction on the accuracy of those methods has not yet been systematically studied.

This paper is devoted to a comparison of three methods for the estimation of selected spatiotemporal gait parameters based on data from a depth sensor, *viz.*:

- a method based on the analysis of the anteroposterior distance between the examined person’s knees, being a variant of the method described in [33];
- a method based on the analysis of the vertical oscillations of the examined person’s centre of mass, being a variant of the method described in [22];
- a method based on the analysis of the horizontal velocity of the examined person’s feet, being a variant of the method described in [20].

The uncertainty of the estimates of selected spatiotemporal gait parameters obtained using these methods was assessed for different depth sensors’ viewing angles and various types of subjects’ clothing.

## 2. Materials and Methods

### 2.1. Studied Data-Processing Methods

#### 2.1.1. Transformation of the Coordinate System

**Figure 2:**
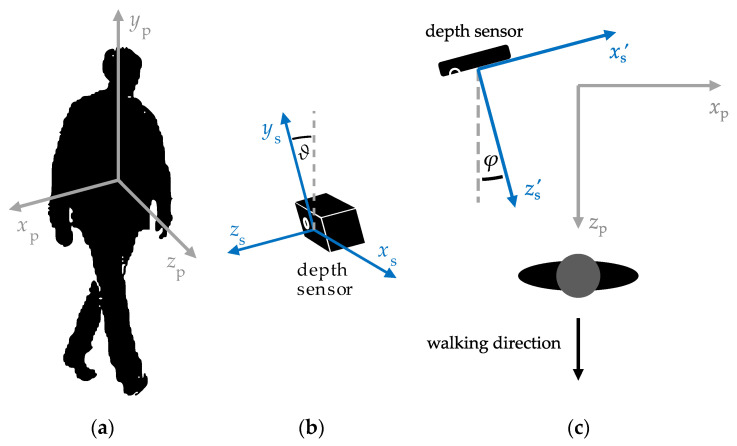
(**a**) The coordinate system (*x*_p_, *y*_p_, *z*_p_) associated with the examined person, in which the *x*_p_ axis corresponds to that person’s medio-lateral axis, the *y*_p_ axis—to that person’s vertical axis and the *z*_p_ axis—to that person’s anteroposterior axis; (**b**) The angle *ϑ* of rotation necessary to make one of the coordinate axes associated with the depth sensor parallel to the examined person’s vertical axis; (**c**) The angle *φ* of rotation necessary to make one of the coordinate axes associated with the depth sensor parallel to the examined person’s walking direction.

The data acquired using a depth sensor represent estimates of the three-dimensional position in a coordinate system relative to the sensor’s internal structure. On the other hand, the studied methods for estimation of spatiotemporal gait parameters, described in subsequent sections of this study, involve processing estimates of the positions of body parts—such as the knees or feet—either along the body’s antero-posterior axis or its vertical axis. Therefore, before further processing, two rotations of the depth sensor’s coordinate system are necessary: a rotation that places one of the coordinate axes parallel to the examined person’s vertical axis and a rotation that places one of the remaining coordinate axes parallel to the person’s walking direction (Figure 2).

The angle *ϑ* between the sensor’s *y*_s_ axis and the examined person’s *y*_p_ axis (Figure 2b) can be estimated by identifying the ground plane, e.g., using the RANSAC algorithm [44]. Algorithms for this purpose are implemented in popular devices comprising depth sensors such as the Microsoft Kinect devices [45]. The corresponding rotation of the coordinate system can be described by the following formula:

(1) $$\left[\begin{array}{c} {x^{\prime }}_{s}\\ {y^{\prime }}_{s}\\ {z^{\prime }}_{s} \end{array}\right]=\left[\begin{array}{ccc} 1 & 0 & 0\\ 0 & \cos\vartheta & \sin\vartheta \\ 0 & -\sin\vartheta & \cos\vartheta \end{array}\right]\left[\begin{array}{c} x_{s}\\ y_{s}\\ z_{s} \end{array}\right]$$

where *x*_s_, *y*_s_ and *z*_s_ denote coordinates along the sensor’s axes shown in Figure 2. The angle *φ* between the sensor’s ${z^{\prime }}_{s}$ axis and the examined person’s walking direction (Figure 2c) can be estimated by fitting the horizontal trajectory of that person’s centre of mass with a straight line. The position of the examined person’s centre of mass can be approximated by an estimate of the position of that person’s spine base, which—in turn—can be obtained using a typical algorithm for localisation of anatomical landmarks in human silhouettes in depth images.

The corresponding rotation of the coordinate system can be described by the following formula:

(2) $$\left[\begin{array}{c} x_{p}\\ y_{p}\\ z_{p} \end{array}\right]=\left[\begin{array}{ccc} \cos\varphi & 0 & \sin\varphi \\ 0 & 1 & 0\\ -\sin\varphi & 0 & \cos\varphi \end{array}\right]\left[\begin{array}{c} {x^{\prime }}_{s}\\ {y^{\prime }}_{s}\\ {z^{\prime }}_{s} \end{array}\right]$$

#### 2.1.2. KD Method

The data processing method called the KD method (referring to the acronym of “knee distance”) is based on the observation that during walking, the local maxima of the distance between the knees, measured along the anteroposterior axis, approximately coincide with the foot contact (FC) moments (i.e., the moments when the feet touch the floor) [46].

The coordinates of the positions of the examined person’s knees can be estimated from depth images using a general-purpose algorithm for locating anatomical landmarks. Such algorithms are implemented in the Microsoft Kinect devices. Similar algorithms, applicable to other devices comprising depth sensors, are available commercially. Alternatively, these coordinates can be estimated using a dedicated algorithm for locating knees. Such an algorithm may involve extracting human silhouettes from depth images and identifying the parts of these silhouettes which correspond to the knees. That identification can be based on the observation that the knees are located at about 0.26 body height [46].

**Figure 3:**
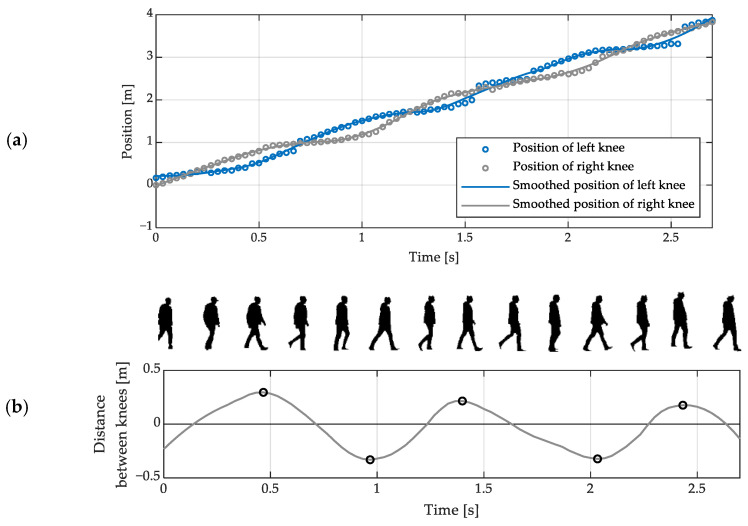
(**a**) Exemplary estimates of anteroposterior positions of knees during walking, obtained using a depth sensor, and the results of their smoothing using a Savitzky-Golay filter; (**b**) An exemplary dependence of the anteroposterior distance between a walking person’s knees on time, with its local extrema indicated with circles and the silhouettes of that person extracted from the corresponding depth images.

The estimation of spatiotemporal gait parameters using the KD method involves the following sequence of operations:

1. smoothing of the sequences of knee position coordinates, e.g., using a Savitzky-Golay filter (Figure 3a);
2. computation of the anteroposterior distance between the knees according to the following formula:

(3) $$d_{n}\;=\;{\hat{x}}_{\mathrm{KR},n}-{\hat{x}}_{\mathrm{KL},n}\in \mathbb{R},\;n=1,\ldots ,N,$$

where ${\hat{x}}_{\mathrm{KL},1},\ldots ,{\hat{x}}_{\mathrm{KL},N}$ denote the smoothed estimates of the anteroposterior position of the left knee, ${\hat{x}}_{\mathrm{KR},1},\ldots ,{\hat{x}}_{\mathrm{KR},N}$—of the right knee, and *N* is the number of depth images contained in the data set under analysis;

3. detection of the left and right FC moments by identifying the local minima and maxima, respectively, of the sequence $d_{1},\ldots ,d_{N}$ (Figure 3b);
4. estimation of the step and stride times based on the detected FC moments;
5. estimation of the step and stride lengths and widths based on estimates of positions of feet at the detected FC moments.

The positive values of the elements of the sequence *d*_1_, …, *d_N_* correspond to the time intervals when the right knee is ahead of the left one. Its negative values, on the other hand, correspond to the time intervals when the left knee is ahead of the right one.

The left step time is defined as the average time which elapses from a right FC moment to the next left FC moment [47]. It can be estimated as the average time from a local minimum of the sequence *d*_1_, …, *d_N_* to its subsequent local maximum. The right step time—defined analogously for the right foot—can be estimated as the average time from a local maximum of that sequence to its subsequent local minimum. Stride time is the average time which elapses between two consecutive FC moments of the same foot [47]. It can be estimated as the average time between successive local minima of that sequence or as the average time between its successive local maxima. One of these options should be chosen so as to use as much of the available data as possible. For example, in the case illustrated in Figure 3, the time between the local maxima should be considered because that particular dataset represents two full strides which start with right FC moments and only one full stride which starts with a left FC moment.

The positions of the feet, estimated at the FC moments detected according to the above-described procedure, can be used to estimate the left and right stride length, step length, stride width and step width according to standard definitions [47].

#### 2.1.3. CH Method

The data-processing method, called the CH method (referring to the acronym of “centre height”) is based on the observation that during gait, the local minima of the height of the centre of mass approximately coincide with the FC moments [48]. On the other hand, its local maxima approximately coincide with the mid-swing (MS) moments, i.e., the moments when the anteroposterior positions of both feet are equal. Furthermore, at the MS moments, the centre of mass is also approximately aligned with the feet along the anteroposterior axis. As in the case of the KD method, the coordinates of the position of the examined person’s centre of mass can be obtained using a general-purpose algorithm for processing depth images or a dedicated one. The latter may involve the extraction of the examined person’s silhouettes from depth images and the identification of the centre of each silhouette [49].

**Figure 4:**
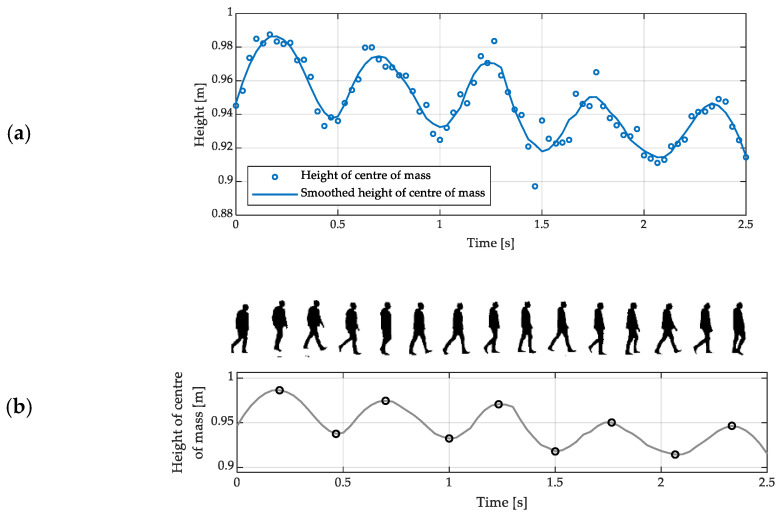
(**a**) Exemplary estimates of the height of the examined person’s centre of mass during walking, obtained using a depth sensor, and the results of their smoothing using a Savitzky-Golay filter; (**b**) an exemplary dependence of the height of a walking person’s centre of mass on time, with its local extrema indicated with circles and the corresponding silhouettes of that person extracted from the depth images.

The estimation of spatiotemporal gait parameters using the CH method involves the following sequence of operations:

1 smoothing of the sequences of coordinates of the height of the examined person’s centre of mass, e.g., using a Savitzky-Golay filter (Figure 4a);
2 identification of the local minima and maxima of that height (Figure 4b);
3 estimation of the mean step and stride time based on the detected FC moments;
4 estimation of the mean step and stride length based on the detected MS moments.

In addition to the oscillations expected, the exemplary estimates of the height of the examined person’s centre of mass, shown in Figure 4, seem to follow a decreasing trend; it is a consequence of the imperfect identification of the vertical axis at the preprocessing stage (*cf.* Section 2.1.1).

The analysis of the vertical oscillations of the centre of mass does not allow for distinguishing left FC moments from right ones. The mean step time, irrespective of the side, can be estimated as the average time between consecutive local minima of the height of the centre of mass. The stride time can be estimated as the average time between every second local minimum of the height of the centre of mass. The mean step length, irrespective of the side, can be estimated as the average distance between the positions of the centre of mass along the antero-posterior axis at consecutive maxima of its height. The stride length can be estimated as the average distance between the positions of the centre of mass along the antero-posterior axis at every second maximum of its height.

#### 2.1.4. FV Method

**Figure 5:**
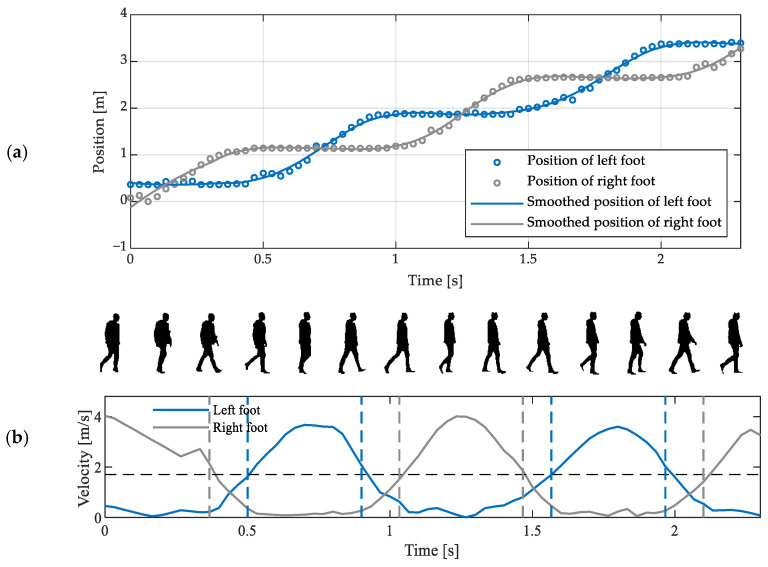
(**a**) Exemplary estimates of anteroposterior positions of a walking person’s feet, obtained using a depth sensor, and the results of their smoothing using a Savitzky-Golay filter; (**b**) Exemplary dependences of the horizontal velocity of a walking person’s feet on time, with the empirically selected threshold value indicated by the horizontal dashed line, the detected FO and FC moments indicated by the vertical dashed lines, and the silhouettes of that person extracted from the corresponding depth images.

The data processing method called the FV method (referring to the acronym of “foot velocity”) is based on the observation that during walking, the horizontal speed of a foot is close to zero during the stance phase and larger during the swing phase. The coordinates of the positions of the examined person’s feet can be estimated from depth images using a general-purpose algorithm locating anatomical landmarks. The estimation of spatiotemporal gait parameters using the FV method involves the following sequence of operations:

1. smoothing of the coordinates of the positions of feet, e.g., using a Savitzky-Golay filter (Figure 5a);
2. estimation of the horizontal velocity of the feet by numerical differentiation of the sequences of position estimates;
3. comparison of that velocity with an empirically selected threshold value;
4. detection of the FC moments and the foot-off (FO) moments (i.e., the moments when a foot is lifted off the floor) by identifying the moments when velocity falls below or rises above—respectively—the aforementioned threshold value (Figure 5b);
5. estimation of selected spatiotemporal gait parameters based on the detected FC and FO moments and the estimates of positions of feet.

The results of the detection of the left and right FO and FC moments, together with the corresponding estimates of the positions of feet, allow for estimating the following spatiotemporal gait parameters according to their standard definitions [47]:

- left and right step time,
- left and right step length,
- stride time,
- stride length,
- left and right swing time,
- left and right stance time,
- double-support time,
- step width.

### 2.2. Methodology of Experimentation

Two sets of data were collected in order to assess the uncertainty of the estimates of spatiotemporal gait parameters obtainable using the three data processing methods described in Section 2.1. The data were collected using a Microsoft Kinect v2 device. Among various types of devices comprising depth sensors, the Kinect v2 device seems to be the one most commonly considered in research studies related to health care applications of such sensors (*cf.* the review of literature in Section 1), although it has not been produced since 2017. The use of Kinect v2 is convenient because this device provides estimates of positions of anatomical landmarks without the need to purchase additional software packages. The reference data were collected using a 2-m long Zebris FDM platform.

**Figure 6:**
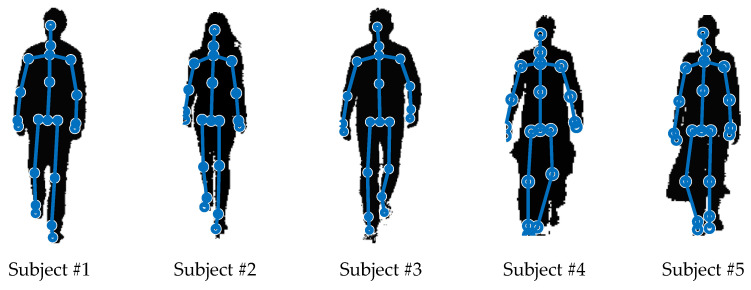
Examples of the results of detecting the silhouettes and locating the anatomical landmarks of the 5 people participating the experiment.

The spatiotemporal parameters were estimated for 5 subjects: 3 women and 2 men, aged 22–45, free from gait disturbances. Subjects #1–#3 wore typical trousers, Subject #4—wide-leg trousers, and Subject #5 wore a skirt. Some anthropometric information about the subjects is collected in Table 1. An exemplary silhouette of each subject is shown in Figure 6. It can be seen in Figure 6 that the localisation of knees, ankles and feet is hindered in the case of these particular silhouettes of Subjects #4 and #5 because of their clothing.

**Figure 7:**
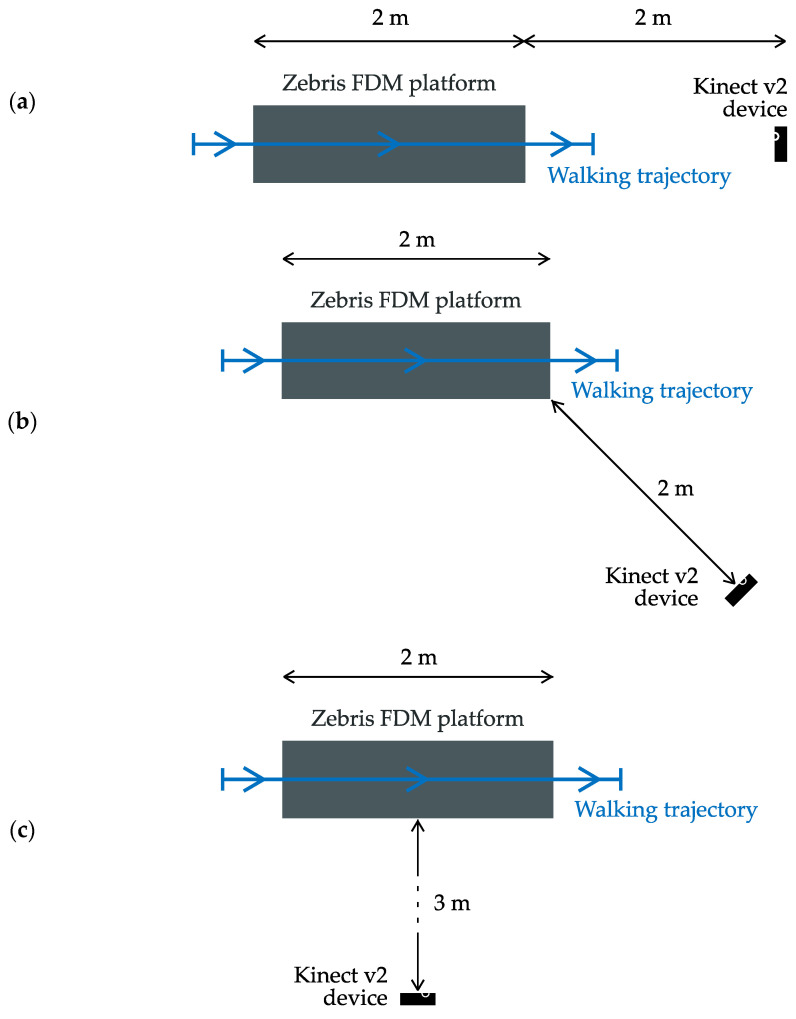
Configurations of the devices used in the experiments in which the angle *φ* between the depth sensor’s line of sight and the walking trajectory is approximately (**a**) 180°, (**b**) 135° and (**c**) 90°.

Three experiments were conducted involving three different angles between the depth sensor’s line of sight and the walking direction (Figure 7):

- depth sensor’s line of sight parallel to the walking direction (*φ* ≅ 180°);
- depth sensor’s line of sight diagonal to the walking direction (*φ* ≅ 135°);
- depth sensor’s line of sight perpendicular to the walking direction (*φ* ≅ 90°).

All 5 subjects took part in the first two of the experiments listed above, whereas only Subjects #1 and #2 took part in the last one.

The time intervals, covering each of the subjects’ passages across the Zebris FDM platform, have been identified by visually inspecting the sequences of depth images. The subsequences of depth images corresponding to those time intervals have been extracted from the data set. Those subsequences have been processed offline using software developed by the authors. The implementation of the Savitzky-Golay filter available in the MATLAB function *smooth* has been used for smoothing the estimates of positions of the knees, feet and centre of mass [50]. In the conducted experiments, the MATLAB function smooth has yielded better results than the function sgolay and another implementation of the Savitzky-Golay filter, developed by the authors of this paper. For this reason, in the case of the FD method, the sequences of foot position estimates have been first smoothed and then differentiated using the central-difference method rather than using a differentiating Savitzky-Golay filter. The MATLAB functions *islocalmin* and *islocalmax* have been used for the detection of local extrema [51,52]. All tunable parameters of the data-processing algorithms have been optimised empirically.

According to the examination procedure associated with the Zebris FDM platform, the subjects’ passages across the tensometric platform have been grouped into triplets. In each experiment, each subject made three such triplets of passages. Within each triplet of passages, the estimates of spatiotemporal gait parameters have been averaged. The estimates of those parameters, obtained using the Zebris FDM platform, have been used as reference values. For each spatiotemporal gait parameter under analysis, the following indicators of uncertainty have been computed:

- the mean error *ME*, defined in the following way:

(4) $$ME\;\equiv \;\frac{1}{M}\sum_{m\;=\;1}^{M}\left({\hat{p}}_{m}\;-\;{\dot{p}}_{m}\right)$$

- the standard deviation of errors *SDE*, defined in the following way:

(5) $$SDE\;\equiv \;\sqrt{\frac{1}{M-1}\sum_{m\;=\;1}^{M}\left({\hat{p}}_{m}\;-\;\bar{p}\right)}\;\mathrm{with}\;\bar{p}\;\equiv \;\frac{1}{M}\sum_{m\;=\;1}^{M}{\hat{p}}_{m}$$

- the mean absolute value of the relative errors *MARE*, defined in the following way:

(6) $$MARE\;\equiv \;\frac{1}{M}\sum_{m\;=\;1}^{M}\left|\frac{{\hat{p}}_{m}-{\dot{p}}_{m}}{{\dot{p}}_{m}}\right|\cdot 100\%$$

where:

- *p* denotes a given spatiotemporal gait parameter under analysis—such as the step length or time;
- ${\hat{p}}_{1},{\hat{p}}_{2},\ldots {\hat{p}}_{M}$ denote the estimates of the parameter *p*, based on the data from the depth sensor;
- ${\dot{p}}_{1},{\dot{p}}_{2},\ldots ,{\dot{p}}_{M}$ denote the reference values of the parameter *p*, obtained using the Zebris FDM platform;
- *M* is the number of obtained estimates of the parameter *p*, i.e., the number of triplets of the subjects’ passages across the tensometric platform.

## 3. Results

This section is devoted to the assessment of the accuracy of the estimates of the step time and length, based on the results of the experiments described in Section 2.2. Complete results of these experiments, including the indicators of accuracy of other gait parameters, are presented in Appendix A.

**Figure 8:**
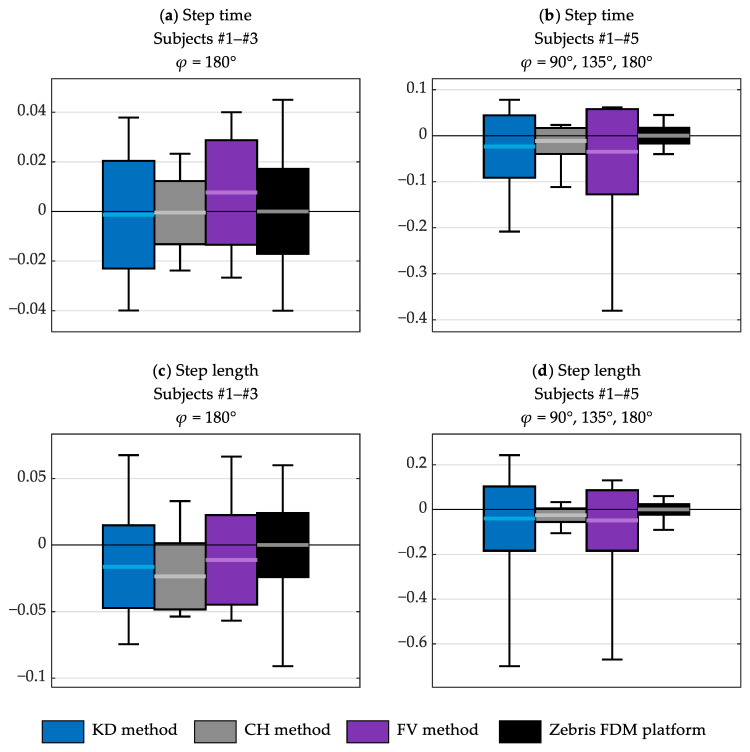
The distribution of the errors of the estimates of the step time (**a**,**b**) and step length (**c**,**d**), obtained using the studied data processing methods; the figures on the left (**a**,**c**) present the results obtained for subjects wearing typical trousers, walking towards the depth sensor; the figures on the right (**b**,**d**) present the results obtained for all subjects in all experiments; the horizontal lines indicate the *ME* values; the height of each box equals the *SDE* value; the whiskers indicate the maximum and minimum errors; the black boxes represent the dispersion of the reference values obtained using the Zebris FDM platform.

Figure 8 shows a comparison of the results obtained using the studied data processing methods. It presents the values of the indicators *ME*, *SDE* and the minimum and maximum errors of the estimates of the step time and length. The results are collected into two sets:

- those obtained only for Subjects #1–#3 (i.e., those who wore typical trousers) and only in the experiments in which *φ* = 180° (i.e., in which the subjects walked towards the depth sensor along its line of sight);
- those obtained for all subjects in all experiments (i.e., for three different angles *φ* between the walking direction and the depth sensor’s line of sight: 90°, 135° and 180°).

The dispersion of the reference values, obtained using the Zebris FDM platform, is also shown in Figure 8. This dispersion was quantified by comparing each reference value to the average reference value of the given parameter obtained for the given subject, and computing the standard deviation of the differences, the maximum difference, and the minimum difference. This dispersion is caused not only by the measurement uncertainty of the Zebris FDM platform, but also by the fact that gait was not perfectly consistent in repeated passages. Such dispersion can also be expected to appear in practical applications related to the analysis of natural overground gait.

It can be seen in Figure 8 that for Subjects #1–#3 and *φ* = 180°, the dispersion of the errors of the estimates of the step time and length is similar to the dispersion of the reference values (Figure 8a,c). It is significantly larger for the KD and FV methods when different values of *φ* and subjects with different clothes are considered (Figure 8b,d). For the CH method, it is somewhat larger in those cases, but the difference is smaller than for the other methods. The step length tends to be underestimated (*cf.*, the horizontal lines in Figure 8c,d), but, on the whole, the mean errors of the estimates of the step time and length are small if compared to their standard deviations.

**Figure 9:**
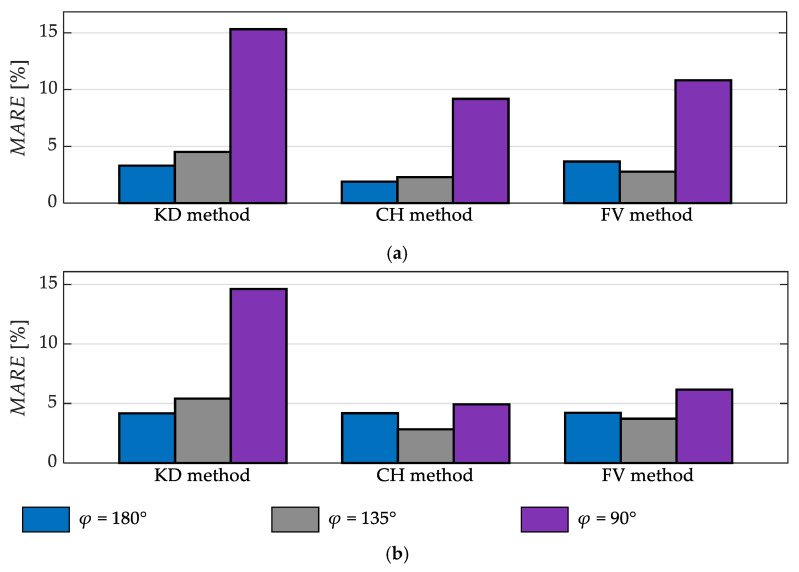
The values of *MARE* obtained for the step time (**a**) and step length (**b**) for Subjects #1–#3 for different angles *φ* between the walking direction and the depth sensor’s line of sight.

Figure 9 shows a comparison of the results obtained for different values of the angle *φ* between the walking direction and the depth sensor’s line of sight. It presents the values of the indicator *MARE* of the estimates of the step time and length obtained for Subjects #1–#3.

It can be seen in Figure 9 that for subjects wearing typical trousers the accuracy of the estimates of the step time and length is similar, regardless of whether the depth sensor’s line of sight is parallel or diagonal to the walking direction (*φ* = 180° or 135°). On the other hand, the step time estimates are significantly less accurate when the depth sensor’s line of sight is perpendicular to the walking direction (*φ* = 90°). The step length estimates obtained using the KD method are also much less accurate for *φ* = 90°. Those obtained using the CH and FV methods are only slightly less accurate in that case.

**Figure 10:**
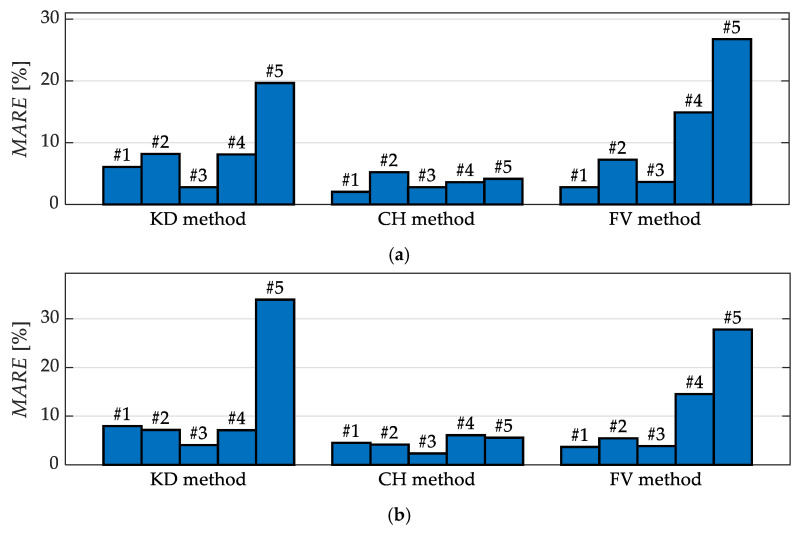
The values of *MARE* obtained for the step time (**a**) and step length (**b**) for *φ* = 90°, 135°, 180° for each subject; the numbers above each bar indicate the subject.

Figure 10 shows a comparison of the results obtained for different subjects. It presents the values of the indicator *MARE* of the estimates of the step time and length for all three values of the angle φ = 90°, 135°, 180°.

It can be seen in Figure 10 that the accuracy of the estimates of the step time and length, obtained using the KD method, is quite similar for subjects wearing typical trousers (#1–#3) and the subject wearing wide-leg trousers (#4), but it is significantly worse for the subject wearing a skirt (#5). The estimates obtained using the CH method have similar accuracy for all subjects. Those obtained using the FV method are significantly less accurate for Subject #4 and even less accurate for Subject #5.

## 4. Discussion

Among the three studied data-processing methods, the FV method allows for the most detailed characterisation of the gait. It enables the determination of the initial-contact moments and the toe-off moments, thus making possible the estimation of the durations of the phases of the gait cycle. The CH method, on the other hand, provides the least information: the left and right steps cannot be distinguished based on the vertical oscillations of the examined person’s centre of mass. It seems that the informativity of the CH method could be increased by supplementing it with the analysis of the lateral (i.e., left-right) position, velocity or acceleration of the centre of mass, but this possibility has not been considered in the study reported herein. The KD method provides more information than the CH method, but less than the FV method.

The results of the conducted experiments indicate that in the case of subjects wearing typical trousers and walking towards the depth sensor (i.e., Subjects #1–#3, *φ* = 180°), all three studied data-processing methods yield quite satisfactory results: the bias of the estimates of spatiotemporal gait parameters is small, and their dispersion is similar to that of the reference values obtained using the Zebris FDM platform (Figure 8a,c). In these conditions, the accuracy of all three methods is similar, with the CH method yielding results slightly less dispersed than the KD and FV methods. Thus, all three studied methods seem to be applicable in gait analysis systems designed to operate in settings in which the subjects’ clothing and walking direction can be controlled to some extent, e.g., in such systems aimed at supporting diagnostic procedures performed at health care facilities. These observations conform with some previously published ones [18,53,54]. The dispersion of the estimates of spatiotemporal gait parameters is partially caused by the imperfection of the data and methods for their processing, and partially—by the natural variability of the human gait. That variability is also reflected by the non-zero dispersion of the reference values.

In the case of subjects wearing different types of clothing and walking in different directions (i.e., Subjects #1–#5; *φ* = 90°, 135°, 180°), the errors corrupting the estimates of spatiotemporal gait parameters, obtained using the KD method and the FV method, are significantly larger (Figure 8b,d). The estimates obtained using the CH method, on the other hand, are corrupted with errors of similar magnitude as in the previous case. The latter method seems, therefore, to have a better applicability potential in systems for in-home monitoring. The accuracy of its results in the various tested conditions is comparable to that of the reference Zebris FDM platform. It only allows, however, for estimating the step time, stride time, step length and stride length—without distinguishing the left and right sides of the body. Further research seems necessary to develop a data-processing method as robust as the CH method, but capable of providing more detailed results of gait analysis.

The depth sensor’s viewing angle has a non-negligible influence on the results of gait analysis, obtainable using the studied data-processing methods. This influence is most pronounced in the case of the KD method (Figure 9): the relative errors of the estimates of spatiotemporal gait parameters are somewhat larger when the subject is observed at the diagonal angle (i.e., *φ* = 135°) and very large when he or she is observed from the side (i.e., *φ* = 90°). These large errors are probably caused by the fact that the antero-posterior position of the knees is easier to estimate when the front of the subject’s silhouette is visible in the depth images. When the subject is observed from the side, one of his or her knees is occluded from time to time. Perhaps, better results could be obtained for *φ* = 90° if the position of the knees were estimated using a dedicated algorithm for processing depth images, instead of a general-purpose algorithm for localisation of anatomical landmarks, but this possibility was not considered in the study reported herein. The originators of this data-processing method considered placing the depth sensor behind a treadmill (i.e., *φ* = 0°) [33].

In the case of the CH method, the estimates of the step time are significantly less accurate for *φ* = 90° than for other viewing angles (Figure 9). This observation indicates that local minima of the height of the centre of mass are more difficult to accurately detect when the subject is observed from the side. On the other hand, the estimates of step length, obtained using the CH method, are comparably accurate for all considered values of *φ*.

In the case of the FV method, the estimates of both the step time and the step length are the most accurate for *φ* = 135° and the least accurate for *φ* = 90°. In the latter case, the errors are large probably because of one of the subject’s feet periodically occluding the other one.

The results of the conducted experiments show that the subject’s clothing may also affect the results of gait analysis (Figure 10). In the case of the KD method, significantly worse results were obtained for Subject #5—the one who wore a skirt—than for other subjects. These results indicate that the localisation of knees based on depth sensor data is hindered when the subject is wearing a skirt, but this is not the case for wide-leg trousers: the results obtained for Subject #4 (who wore such wide-leg trousers) are similar to those obtained for Subject #2 (who wore typical trousers). The results obtained using the CH method are quite similar for all the subjects, which indicates that this method is insensitive to the subject’s clothing. The FV method, on the other hand, is the most sensitive to the subject’s clothing: its results are significantly worse for Subject #4 (who wore wide-leg trousers) than for Subjects #1–#3, and even worse for Subject #5 (who wore a skirt).

The experimental results described above indicate that, in certain conditions, reliable results of spatiotemporal gait analysis can be obtained using the Kinect v2 depth sensor. The CH method can be recommended for the analysis of gait of persons wearing different types of clothing, but if detailed characterisation of gait is sought, then further research is required to develop a data-processing method which would combine the robustness of the CH method and the informativeness of the FV method. None of the studied data-processing methods is capable of providing accurate estimates of spatiotemporal gait parameters of subjects observed from the side (*φ* = 90°), so further research is needed to develop a method useful in that setting. The implementation of a dedicated algorithm for processing raw depth images, instead of using a general-purpose algorithm for localisation of anatomical landmarks, seems a good starting point for such research.

The experiments reported herein were subject to certain limitations. Five subjects took part in these experiments for *φ* = 180° and *φ* = 135°, and only two subjects—for *φ* = 90°. Furthermore, all the subjects were persons of working age, free from gait disorders. The presented experimental findings need, therefore, to be validated using more data in order to corroborate the usefulness of the studied methods for the analysis of gait of elderly people and people suffering from gait disorders.

The development of more sophisticated data processing methods—in particular, methods based on a fusion of information obtained using the three methods studied in this paper—may allow for obtaining more accurate and robust estimates of various useful spatiotemporal gait parameters independently of the subject’s clothing and walking direction. The development of reliable gait analysis systems based on depth sensors—in particular, those dedicated to in-home monitoring settings—seems desirable because such systems have certain advantages over the currently available gait analysis systems based on other technological solutions. The optoelectronic motion-capture systems are much more expensive than depth sensors and they need to be installed in quite large rooms, which makes their application for in-home monitoring impractical. Platforms and treadmills equipped with force sensors, relatively common in clinical facilities and laboratories, are also more expensive and less convenient for in-home use than depth sensors, and they cannot serve for estimating the angles in the ankle, knee and hip joints—which is reportedly feasible using depth sensors [55]. Gait analysis systems based on wearable sensors are proliferating [17], but the need to wear a device on the body or clothes may be considered cumbersome by the potential users of such systems [56]. Furthermore, a system for in-home monitoring of the elderly, based on a wearable device, may become useless if its user forgets to recharge or wear the device or decides not to use it.

## 5. Conclusions

The findings of this study can be summarised as follows:

- The results of gait analysis based on data from a Kinect v2 device can be comparable in accuracy to those based on data from a Zebris FDM platform if the subject is wearing typical trousers and walking toward that device. In such a case, the FV method allows for the most detailed characterisation of human gait.
- The angle between the walking direction and the depth sensor’s line of sight significantly affects the accuracy of the estimates of the spatiotemporal gait parameters, obtained using all three studied data-processing methods.
- The subject’s clothing significantly affects the accuracy of the gait analysis results obtained using the FV method and the KD method, but not the CH method.
- The practical advantages and disadvantages of the studied data processing methods, identified based on experimental results described above, may serve as a basis for further research aimed at developing more versatile methods for spatiotemporal gait analysis, dedicated to in-home monitoring systems.

The authors’ plans for future research include:

- the development and testing of other methods for processing data from depth sensors, aimed at obtaining more accurate and robust estimates of spatiotemporal gait parameters;
- conducting more experiments aimed at testing the usefulness of gait analysis systems based on depth sensors in clinical practice and in systems for in-home monitoring of the elderly.

## Figures and Tables

**Table 1:** Anthropometric data about the subjects and relevant information about their clothing.

Subject	Age [Years]	Gender	Weight [kg]	Height [cm]	Clothing
#1	33	m	85	188	typical trousers
#2	22	f	58	163	typical trousers
#3	25	m	82	175	typical trousers
#4	42	f	74	165	wide-leg trousers
#5	45	f	58	168	skirt

## Data Availability

The data presented in this study are available on request from the corresponding author.

## Appendix A

This Appendix contains all the obtained experimental results. The reference values obtained using the Zebris FDM platform are shown in Table A1. The values of the indicators of accuracy, defined in Section 2.2, obtained for the 3 studied data-processing methods described in Section 2.1, are shown in Table A2, Table A3 and Table A4. These values have been grouped according to:

- the data processing method: KD (Table A2), CH (Table A3) and FV (Table A4);
- the angle *φ* between the depth sensor’s line of sight and the walking direction;
- the subjects whose gait was analysed: Subjects #1–#3, who wore typical trousers, and, separately, Subjects #4 and #5, who wore wide-leg trousers and a skirt, respectively.

**Table A1:** Means and standard deviations (std) of the reference values of selected spatiotemporal gait parameters, obtained using the Zebris FDM platform.

Parameter	Subject #1		Subject #2		Subject #3		Subject #4		Subject #5	
	Mean	Std	Mean	Std	Mean	Std	Mean	Std	Mean	Std
Left step time [s]	0.539	0.022	0.517	0.011	0.480	0.022	0.538	0.020	0.528	0.016
Right step time [s]	0.536	0.013	0.505	0.015	0.485	0.016	0.545	0.028	0.517	0.021
Left step length [cm]	73.1	2.8	71.6	1.9	68.2	2.6	66.1	0.9	65.1	3.2
Right step length [cm]	75.4	1.6	70.4	1.6	65.8	4.6	64.3	3.4	67.9	1.0
Stride time [s]	1.074	0.033	1.023	0.017	0.965	0.030	1.083	0.035	1.045	0.022
Stride length [cm]	148.5	3.7	142.1	3.3	134.0	5.5	130.3	3.1	133.0	3.8
Left swing time [%]	38.5	1.2	40.4	0.8	36.3	1.6	35.7	1.1	39.0	1.6
Right swing time [%]	37.4	0.3	39.3	0.7	37.7	2.3	38.3	1.3	37.9	1.1
Left stance time [%]	61.5	1.2	59.6	0.8	63.7	1.6	64.3	1.1	61.0	1.6
Right stance time [%]	62.6	0.3	60.7	0.7	62.3	2.3	61.7	1.3	62.1	1.1
Double-support time [%]	24.1	1.4	20.1	1.0	25.3	2.1	26.0	0.5	23.1	1.0
Step width [cm]	12.3	1.1	7.2	0.6	12.8	0.6	15.6	1.2	8.4	1.9

**Table A2:** The indicators of accuracy of the estimates of spatiotemporal gait parameters, obtained using the KD method.

	*φ* = 180°		*φ* = 135°		*φ* = 90°
	Subjects #1–#3	Subjects #4, #5	Subjects #1–#3	Subjects #4, #5	Subjects #1, #2
*ME*					
Left step time [s]	−0.007	−0.016	0.003	−0.073	−0.053
Right step time [s]	0.004	0.004	0.003	−0.137	−0.033
Left step length [cm]	−1.5	−5.1	1.6	10.4	1.1
Right step length [cm]	−1.8	−2.8	−2.2	−33.2	−15.9
Stride time [s]	0.004	−0.016	0.011	−0.150	−0.125
Stride length [cm]	0.8	−4.3	−2.3	−15.3	−19.1
Step width [cm]	7.6	7.6	4.6	−1.2	1.4
*SDE*					
Left step time [s]	0.021	0.040	0.028	0.121	0.125
Right step time [s]	0.022	0.029	0.036	0.062	0.066
Left step length [cm]	2.9	4.3	4.1	9.9	6.2
Right step length [cm]	3.4	3.2	5.1	32.8	13.1
Stride time [s]	0.032	0.058	0.013	0.146	0.140
Stride length [cm]	5.3	5.1	4.7	21.5	14.3
Step width [cm]	0.8	1.9	3.2	2.6	3.9
*MARE*					
Left step time	3.7%	5.0%	3.9%	20.9%	19.1%
Right step time	2.9%	4.0%	5.1%	25.5%	11.6%
Left step length	3.9%	7.7%	5.2%	16.9%	6.8%
Right step length	4.4%	5.1%	5.6%	52.4%	22.5%
Stride time	2.6%	3.4%	1.4%	14.1%	12.8%
Stride length	2.9%	3.6%	2.5%	12.7%	13.7%
Step width	81.8%	67.8%	53.9%	20.6%	26.3%

**Table A3:** The indicators of accuracy of the estimates of spatiotemporal gait parameters, obtained using the CH method.

	*φ* = 180°		*φ* = 135°		*φ* = 90°
	Subjects #1–#3	Subjects #4, #5	Subjects #1–#3	Subjects #4, #5	Subjects #1, #2
*ME*					
Step time [s]	0.000	−0.004	0.003	−0.031	−0.047
Stride time [s]	0.002	−0.012	0.010	−0.057	−0.114
Step length	−2.3	−1.8	−1.4	−4.6	−3.6
Stride length [cm]	−1.9	−3.8	1.3	−4.0	−3.1
*SDE*					
Step time [s]	0.013	0.013	0.015	0.026	0.047
Stride time [s]	0.027	0.025	0.025	0.062	0.081
Step length [cm]	2.5	2.1	2.2	4.3	4.1
Stride length [cm]	3.3	4.4	4.4	8.2	4.0
*MARE*					
Step time	1.9%	2.0%	2.3%	5.8%	9.2%
Stride time	1.9%	2.0%	2.1%	6.0%	11.0%
Step length	4.2%	3.1%	2.8%	8.6%	4.9%
Stride length	2.3%	3.3%	2.7%	6.0%	2.9%

**Table A4:** The indicators of accuracy of the estimates of spatiotemporal gait parameters, obtained using the FV method.

	*φ* = 180°		*φ* = 135°		*φ* = 90°
	Subjects #1–#3	Subjects #4, #5	Subjects #1–#3	Subjects #4, #5	Subjects #1, #2
*ME*					
Left step time [s]	0.003	−0.001	−0.002	−0.251	−0.065
Right step time [s]	0.012	0.012	0.004	−0.141	−0.032
Left step length [cm]	−0.6	−3.2	0.0	−10.7	−1.5
Right step length [cm]	−1.6	−3.9	−0.8	−31.9	−4.9
Stride time [s]	0.016	−0.013	−0.001	−0.415	−0.143
Stride length [cm]	3.6	−4.2	−0.1	−56.2	−8.5
Left swing phase [%]	−1.0	0.9	0.6	0.0	−0.8
Right swing phase [%]	−0.8	−1.4	1.7	−0.4	5.1
Left stance phase [%]	1.0	0.9	0.3	0.1	0.8
Right stance phase [%]	0.8	1.4	−1.7	1.0	−5.1
Double-support phase [%]	2.1	2.0	−1.4	0.5	−3.5
Step width [cm]	7.6	9.0	4.9	0.6	0.7
*SDE*					
Left step time [s]	0.020	0.019	0.016	0.138	0.088
Right step time [s]	0.022	0.040	0.018	0.076	0.049
Left step length [cm]	3.3	3.2	3.5	10.6	1.7
Right step length [cm]	3.5	2.2	2.9	35.8	11.3
Stride time [s]	0.034	0.023	0.042	0.128	0.205
Stride length [cm]	7.8	3.5	3.7	19.5	13.1
Left swing phase [%]	1.7	2.9	3.1	5.2	8.0
Right swing phase [%]	1.1	2.2	2.2	6.1	6.4
Left stance phase [%]	1.7	2.9	3.6	4.2	8.0
Right stance phase [%]	1.1	2.2	2.2	5.3	6.4
Double-support phase [%]	2.2	1.6	3.4	6.4	3.0
Step width [cm]	0.9	1.8	2.4	4.0	3.6
*MARE*					
Left step time	3.0%	2.5%	2.4%	48.4%	13.2%
Right step time	4.3%	6.2%	3.1%	26.3%	8.4%
Left step length	3.8%	5.1%	4.0%	19.2%	2.4%
Right step length	4.6%	5.9%	3.4%	54.4%	10.0%
Stride time	2.8%	1.7%	3.1%	39.0%	14.9%
Stride length	4.6%	3.7%	2.1%	42.7%	6.3%
Left swing phase	4.4%	4.5%	6.1%	12.6%	14.2%
Right swing phase	2.8%	4.9%	5.2%	12.4%	13.8%
Left stance phase	2.8%	2.8%	4.5%	5.7%	9.2%
Right stance phase	1.7%	3.0%	3.1%	6.8%	8.8%
Double-support phase	10.7%	8.2%	11.1%	20.9%	16.6%
Step width	80.7%	80.6%	53.9%	34.0%	29.8%

## References

[1] Montero-Odasso M., Camicioli R. (2020). Falls as a manifestation of brain failure: Gait, cognition, and the neurobiology of falls. Falls and Cognition in Older Persons: Fundamentals, Assessment and Therapeutic Options.

[2] Armand S., Decoulon G., Bonnefoy-Mazure A. (2016). Gait analysis in children with cerebral palsy. Efort Open Rev..

[3] Newman A.B., Simonsick E.M., Naydeck B.L., Boudreau R.M., Kritchevsky S.B., Nevitt M.C., Pahor M., Satterfield S., Brach J.S., Studenski S.A. (2006). Association of long-distance corridor walk performance with mortality, cardiovascular disease, mobility limitation, and disability. JAMA.

[4] Ewins D., Collins T., Taktak A., Ganney P., Long D., White P. (2014). Clinical Gait Analysis. Clinical Engineering.

[5] Maki B.E. (1997). Gait changes in older adults: Predictors of falls or indicators of fear?. J. Am. Geriatr. Soc..

[6] Pieruccini-Faria F., Montero-Odasso M., Hausdorff J.M., Montero-Odasso M., Camicioli R. (2020). Gait variability and fall risk in older adults: The role of cognitive function. Falls and Cognition in Older Persons: Fundamentals, Assessment and Therapeutic Options.

[7] Hausdorff J.M., Cudkowicz M.E., Firtion R., Wei J.Y., Goldberger A.L. (1998). Gait variability and basal ganglia disorders: Stride-to-stride variations of gait cycle timing in Parkinson’s disease and Huntington’s disease. Mov. Disord..

[8] Robinovitch S.N., Feldman F., Yang Y., Schonnop R., Leung P.M., Sarraf T., Sims-Gould J., Loughin M. (2013). Video capture of the circumstances of falls in elderly people residing in long-term care: An observational study. Lancet.

[9] Rubenstein L.Z., Josephson K.R. (2002). The epidemiology of falls and syncope. Clin. Geriatr. Med..

[10] United Nations Department of Economic and Social Affairs—Population Division World Population Prospects. https://population.un.org/wpp/publications/.

[11] Allali G., Launay C.P., Blumen H.M., Callisaya M.L., De Cock A.-M., Kressig R.W., Srikanth V., Steinmetz J.-P., Verghese J., Beauchet O. (2017). Falls, cognitive impairment, and gait performance: Results from the GOOD initiative. J. Am. Med. Dir. Assoc..

[12] Mulas I., Putzu V., Asoni G., Viale D., Mameli I., Pau M. (2021). Clinical assessment of gait and functional mobility in Italian healthy and cognitively impaired older persons using wearable inertial sensors. Aging Clin. Exp. Res..

[13] Bock J.-O., König H.-H., Brenner H., Haefeli W.E., Quinzler R., Matschinger H., Saum K.-U., Schöttker B., Heider D. (2016). Associations of frailty with health care costs—Results of the ESTHER cohort study. BMC Health Serv. Res..

[14] Peetoom K.K.B., Lexis M.A.S., Joore M., Dirksen C.D., De Witte L.P. (2015). Literature review on monitoring technologies and their outcomes in independently living elderly people. Disabil. Rehabil. Assist. Technol..

[15] World Health Organisation Integrated Care for Older People: Guidelines on Community-Level Interventions to Manage Declines in Intrinsic Capacity. https://apps.who.int/iris/handle/10665/258981.

[16] Rudisch J., Jöllenbeck T., Vogt L., Cordes T., Klotzbier T.J., Vogel O., Wollesen B. (2021). Agreement and consistency of five different clinical gait analysis systems in the assessment of spatiotemporal gait parameters. Gait Posture.

[17] Prasanth H., Caban M., Keller U., Courtine G., Ijspeert A., Vallery H., von Zitzewitz J. (2021). Wearable sensor-based real-time gait detection: A systematic review. Sensors.

[18] Clark R.A., Mentiplay B.F., Hough E., Pua Y.H. (2019). Three-dimensional cameras and skeleton pose tracking for physical function assessment: A review of uses, validity, current developments and Kinect alternatives. Gait Posture.

[19] Otte K., Kayser B., Mansow-Model S., Verrel J., Paul F., Brandt A.U., Schmitz-Hübsch T. (2016). Accuracy and reliability of the Kinect Version 2 for clinical measurement of motor function. PLoS ONE.

[20] Ferraris C., Cimolin V., Vismara L., Votta V., Amprimo G., Cremascoli R., Galli M., Nerino R., Mauro A., Priano L. (2021). Monitoring of gait parameters in post-stroke individuals: A feasibility study using RGB-D sensors. Sensors.

[21] Dubois A., Bihl T., Bresciani J.-P. (2019). Automatic measurement of fall risk indicators in timed up and go test. Inform. Health Soc. Care.

[22] Dubois A., Bihl T., Bresciani J.-P. (2021). Identifying fall risk predictors by monitoring daily activities at home using a depth sensor coupled to machine learning algorithms. Sensors.

[23] Guffanti D., Brunete A., Hernando M. (2020). Non-invasive multi camera gait analysis system and its application to gender classification. IEEE Access.

[24] Chaparro-Rico B.D.M., Cafolla D. (2020). Test-retest, inter-rater and intra-rater reliability for spatiotemporal gait parameters using SANE (an eaSy gAit aNalysis systEm) as measuring instrument. Appl. Sci..

[25] Cui X., Zhao Z., Ma C., Chen F., Liao H. A gait character analyzing system for osteoarthritis pre-diagnosis using RGB-D camera and supervised classifier. Proceedings of the World Congress on Medical Physics and Biomedical Engineering.

[26] Burle A.D., Lafayette T.B.D., Fonseca J.R., Teichrieb V., Da Gama A.E.F. Real-time approach for gait analysis using the Kinect v2 sensor for clinical assessment purpose. Proceedings of the 22nd Symposium on Virtual and Augmented Reality (SVR).

[27] Castaño-Pino Y.J., González M.C., Quintana-Peña V., Valderrama J., Muñoz B., Orozco J., Navarro A. Automatic gait phases detection in Parkinson disease: A comparative study. Proceedings of the 42nd Annual International Conference of the IEEE Engineering in Medicine and Biology Society.

[28] Albert J.A., Owolabi V., Gebel A., Brahms C.M., Granacher U., Arnrich B. (2020). Evaluation of the pose tracking performance of the Azure Kinect and Kinect v2 for gait analysis in comparison with a gold standard: A pilot study. Sensors.

[29] Vilas-Boas M.d.C., Rocha A.P., Choupina H.M.P., Cardoso M.N., Fernandes J.M., Coelho T., Cunha J.P.S. (2019). Validation of a single RGB-D camera for gait assessment of polyneuropathy patients. Sensors.

[30] Rocha A.P., Choupina H.M.P., Vilas-Boas M.D.C., Fernandes J.M., Cunha J.P.S. (2018). System for automatic gait analysis based on a single RGB-D camera. PLoS ONE.

[31] Atanasov M., Kampel M. Automated determination of gait parameters using depth based person tracking. Proceedings of the 16th IEEE International Conference on Embedded and Ubiquitous Computing.

[32] Geerse D., Coolen B., Kolijn D., Roerdink M. (2017). Validation of foot placement locations from ankle data of a Kinect v2 sensor. Sensors.

[33] Auvinet E., Multon F., Manning V., Meunier J., Cobb J.P. (2017). Validity and sensitivity of the longitudinal asymmetry index to detect gait asymmetry using Microsoft Kinect data. Gait Posture.

[34] Amini A., Banitsas K., Hosseinzadeh S. A new technique for foot-off and foot contact detection in a gait cycle based on the knee joint angle using Microsoft Kinect v2. Proceedings of the 4th IEEE EMBS International Conference on Biomedical and Health Informatics (BHI).

[35] Xu X., McGorry R.W., Chou L.-S., Lin J.-H., Chang C.-C. (2015). Accuracy of the Microsoft Kinect™ for measuring gait parameters during treadmill walking. Gait Posture.

[36] Hynes A., Czarnuch S., Kirkland M.C., Ploughman M. (2021). Spatiotemporal gait measurement with a side-view depth sensor using human joint proposals. IEEE J. Biomed. Health Inform..

[37] Latorre J., Llorens R., Colomer C., Alcañiz M. (2018). Reliability and comparison of Kinect-based methods for estimating spatiotemporal gait parameters of healthy and post-stroke individuals. J. Biomech..

[38] Valencia-Jimenez N., Leal-Junior A., Avellar L., Vargas-Valencia L., Caicedo-Rodriguez P., Ramirez-Duque A.A., Lyra M., Marques C., Bastos T., Frizera A. (2019). A comparative study of markerless systems based on color-depth cameras, polymer optical fiber curvature sensors, and inertial measurement units: Towards increasing the accuracy in joint angle estimation. Electronics.

[39] Pathegama M.P., Marasinghe D.M., Wijayasekara K., Karunanayake I., Edussooriya C.U.S., Silva P., Rodrigo R. Moving Kinect-based gait analysis with increased range. Proceedings of the IEEE International Conference on Systems, Man, and Cybernetics.

[40] Andre J., Lopes J., Palermo M., Goncalves D., Matias A., Pereira F., Afonso J., Seabra E., Cerqueira J., Santos C. Markerless gait analysis vision system for real-time gait monitoring. Proceedings of the IEEE International Conference on Autonomous Robot Systems and Competitions.

[41] Paulo J., Asvadi A., Peixoto P., Amorim P. (2017). Human gait pattern changes detection system: A multimodal vision-based and novelty detection learning approach. Biocybern. Biomed. Eng..

[42] Wagner J., Morawski R.Z., Mazurek P. (2022). Non-Invasive Monitoring of Elderly Persons: Systems Based on Impulse-Radar Sensors and Depth Sensors.

[43] Dubois A., Charpillet F. (2017). Measuring frailty and detecting falls for elderly home care using depth camera. J. Ambient. Intell. Smart Environ..

[44] Kırcalı D., Tek F.B. Ground plane detection using an RGB-D sensor. Proceedings of the 29th International Symposium on Computer and Information Sciences.

[45] Pterneas V. Floor Detection Using Kinect. https://pterneas.com/2017/09/10/floor-kinect/.

[46] Auvinet E., Multon F., Meunier J. Lower limb movement asymmetry measurement with a depth camera. Proceedings of the 2012 Annual International Conference of the IEEE Engineering in Medicine and Biology Society.

[47] zebris Medical GmbH zebris FDM Software Manual. https://www.zebris.de/fileadmin/Editoren/zebris-PDF-Manuals/Medizin/Software/Alte_Versionen/Manual_zebris_FDM_1.16.x_R1_EN_web.pdf.

[48] Dubois A., Charpillet F. A gait analysis method based on a depth camera for fall prevention. Proceedings of the 36th Annual International Conference of the IEEE Engineering in Medicine and Biology Society.

[49] Mazurek P., Wagner J., Morawski R.Z. (2018). Use of kinematic and mel-cepstrum-related features for fall detection based on data from infrared depth sensors. Biomed. Signal Process. Control..

[50] MathWorks MATLAB Smooth. https://www.mathworks.com/help/curvefit/smooth.html.

[51] MathWorks MATLAB Islocalmin. https://www.mathworks.com/help/matlab/ref/islocalmin.html.

[52] MathWorks MATLAB Islocalmax. https://www.mathworks.com/help/matlab/ref/islocalmax.html.

[53] Steinert A., Sattler I., Otte K., Röhling H., Mansow-Model S., Müller-Werdan U. (2020). Using New Camera-Based Technologies for Gait Analysis in Older Adults in Comparison to the Established GAITRite System. Sensors.

[54] Dubois A., Bresciani J.-P. (2018). Validation of an ambient system for the measurement of gait parameters. J. Biomech..

[55] Vilas-Boas M.d.C., Rocha A.P., Cardoso M.N., Fernandes J.M., Coelho T., Cunha J.P.S. (2021). Supporting the assessment of hereditary transthyretin amyloidosis patients based on 3-D gait analysis and machine learning. IEEE Trans. Neural Syst. Rehabil. Eng..

[56] Thilo F.J.S., Hahn S., Halfens R.J.G., Schols J.M.G.A. (2019). Usability of a wearable fall detection prototype from the perspective of older people: A real field testing approach. J. Clin. Nurs..

